# Resident marine sportfishing effort in the United States varied non-monotonically with COVID policy stringency

**DOI:** 10.1038/s41598-024-60960-4

**Published:** 2024-05-29

**Authors:** Alexander Gordan, David Carter, Christopher Liese

**Affiliations:** 1 ECS Federal, Fairfax, USA; 2 NOAA Southeast Fisheries Science Center, Miami, USA

**Keywords:** Environmental economics, Fisheries

## Abstract

Governments responded to the Covid-19 pandemic with different policies to curtail the spread of the virus. We show how sportfishing levels are related to the stringency of Covid-19 policies. Specifically, we relate the total number of resident sportfishing trips taken each month in each of 16 U.S. states to a state-level index of COVID policy stringency. We model the number of recreational fishing trips taken in each state-month using a fixed effect Poisson regression model with state-specific seasonality and time trends. We estimate separate models for different fishing modes, and find that for fishing trips taken on private boats the number of trips may have increased by approximately 20% at moderate levels of stringency, while at high levels of stringency like those experienced in many states in March and April of 2020, trips may have stayed constant or declined by 10–20%. Similar inverse-U shaped relationships between trips and stringency are found for fishing trips from the shore and from charter boats.

## Introduction

Saltwater angling is a major outdoor activity in the U.S., with expenditures on fishing trips totaling more than $10 billion annually as of 2017^1^. Roughly 4% of Americans participate in saltwater fishing compared with around around 20% for running, the most common outdoor recreation activity^2^. Furthermore, recreational angling can be a substantial component of the fishery, especially in the Southeastern US, where sportfishing accounts for a large share of the harvest of some commercially and culturally significant species^3^.

The COVID-19 pandemic and ensuing policy responses had far-reaching social and economic implications, and marine angling is no exception. Landry et al.^4^ summarize the potential pathways through which COVID-19 and related policy responses may affect outdoor recreation. For recreational fishing the potential pathways are as follows:

1. Site closures may force anglers to cancel trips if, for example, boat ramps or piers were closed^5^.
2. Concern about infection risk may have led people to cancel planned trips or cease to plan new trips^6^.
3. People may have more leisure time due to work-from-home flexibility, reduced commuting time, or job loss^7^.
4. A decrease in substitute leisure activities, especially indoor activities, may have made angling relatively more attractive as a way to spend one’s leisure time^8,9^

The first two pathways imply a decrease in fishing effort while the latter two imply an increase. Our hypothesis regards the net effect of these different mechanisms, and specifically we expect a non-monotonic relationship between fishing effort and stringency. For example, early in the response to the pandemic when lockdowns were common, the effect of site closures and concerns about infection risk would dominate and effort should be relatively low. At other times, however, as lockdowns are relaxed, but restrictions on indoor gatherings and leisure travel remain, effort could increase due to the effects of increased leisure time and lack of substitute activities for that leisure time. A similar type of variation in trip taking behavior could happen over space, i.e. the different levels of COVID-19 policy stringency in different jurisdictions (e.g., states) could contribute to variations in angler trip taking behavior across jurisdictions.

Link et al.^10^ documents interruptions in U.S. marine fishery data collections due to COVID-19 policies such as lockdowns. Many of these interruptions also affected anglers’ access to fishing launch sites. Our focus is on saltwater anglers, but Paradis et al.^11^ found that around ninety percent of freshwater fishing jurisdictions in North America were able to keep fishing open during spring of 2020. In a national survey of U.S. anglers, most respondents experienced some access restrictions, but nearly all respondents reported that they did not think that recreational fishing was unsafe during the early stages of the pandemic in 2020^8^. The same study found that anglers reported very little change in the number of trips they took relative to the same period in years past. However, this is self-reported changes for Spring 2020 averaged over various levels of COVID-19 policy stringencies. Actual changes in effort over a longer period of time and range of COVID-19 policy stringencies may be different.

Using data from fish finding devices in Europe Audzijonyte et al.^12^, find substantial increases in fishing activity during the Spring of 2020. In Denmark Gundelund and Skov^13^, report a 20% bump in angling license sales, with the newly registered anglers being on average younger and more urban, however with lower catch rates as well. Hook et al.^14^ use panel survey data from the UK and find that marine angling decreased in Spring of 2020. Ryan et al.^15^ present a variety of evidence from Western Australia including surveys of licensed anglers and boat ramp camera footage, and hypothesize that travel restrictions impacted especially urban anglers. We contribute to this literature by applying the best available data on US marine angling activity to analyze the impact of COVID stringency on fishing effort.

We use data on the estimated monthly number of saltwater fishing trips from states on the U.S. east coast and Gulf of Mexico along with a monthly, state-level measure of COVID-19 policy stringency to examine the relationship between the stringency of COVID-19 policy and the level of marine angling activity. Our results suggest that the relationship is non-monotonic, whereby the number of fishing trips increases at moderate levels of COVID-19 policy, but declines at higher levels. This finding is important to understanding how people respond to measures aimed at containing virus spread and can help in planning during future pandemics, which will be more likely in the future than they have been in the past due to factors including climate change^16^. More modestly, our research suggests that even events less severe than a pandemic, such as an especially intense flu season, may have impacts on recreational fishing effort if they reduce the availability or attractiveness of substitute leisure activities. This has implications for the management of the fisheries, which are currently regulated in an open access fashion such that these impacts may challenge the ability of the management system to achieve optimum yield.

## Methods

### Data

To conduct our analysis, we construct a panel dataset at the state-month level spanning 2017 through 2021 for 16 US states, by merging two primary data sources: (1) the Marine Recreational Information Program (MRIP), which provides the necessary angler survey data to construct monthly estimates of the fishing activity in each state, providing our dependent variable, and (2) the Oxford COVID Government Response Tracker (OxCGRT), which provides fine-grained (daily) variation in the level of COVID policy stringency in each of the US states, which we collapse to the monthly level to provide our key independent variable. Below, we describe in greater detail these two major data sources, as well as state population data used in the analysis, and our methodology for processing these data sources to produce our panel dataset.

#### MRIP

The MRIP program oversees survey research to monitor the level of marine angling activity in a consistent fashion throughout US fisheries (we discuss the structure of these surveys in greater detail below). The program consists of 2 main data collections. The first data collection we use from MRIP is the Fishing Effort Survey (FES), a mail survey which asks anglers to recall how many trips they have been on recently, in order to get information on the total level of fishing activity. The other data collection is the Access Point Angler Intercept Survey (APAIS), an intercept survey that captures trip-level data about catch, angler attributes, and trip details such as area fished.

We use the MRIP data for the 16 states along the Gulf and Atlantic coasts in which it is available. The MRIP program publishes estimates of fishing activity for 2-month periods referred to as waves, but rather than use these estimates we use the publicly-available microdata files to estimate total resident trips in each state-month. These microdata are anonymized versions of the completed APAIS data and are thus trip-level data, and they are augmented with survey weights derived from the FES data that allow for the construction of estimates that are representative of all trips covered by the MRIP program. The primary unit of measurement for our aggregated summary of the MRIP data is the angler-trip (hereafter abbreviated simply to trip), representing the total number of distinct trips multiplied by the average party size of those trips.

The FES mail survey uses a sampling frame of all residential addresses in the states it operates in, obtained from the United States Postal Service. From this frame, stratified random sampling of addresses is conducted, stratifying both by proximity to the coast, as well as whether the household is matched to a database of fishing licenses, so as to most effectively sample households which are likely to have engaged in recreational fishing. Households receive a survey quetionnaire at the end of a given 2-month period, for recall within that period, and the mailing includes a cover letter describing the purpose of the survey as well as a $2 cash incentive and a postage-paid return envelope. The response rate to the FES is approximately 35%, and post-stratification is conducted using population data from the Census’ American Community Survey to make the estimated number of trips representative of the overall population.

We assemble monthly observations from 2017 through 2021 for three different modes of fishing: private boats, charter boats, and shore fishing. It is important to separately consider these different modes of fishing in our analyis because they represent different angling populations, different species being fished for, and in the case of the charter mode is managed under different rules. With 5 years, 12 months in each year and 16 states, the data set for each mode could potentially have 960 observations. However, the data collection is not conducted for certain state-months for which there is known to be minimal fishing activity for a given mode, which are typically the winter months in colder states. According to the MRIP Data User Handbook and the MRIP Survey Design Manual, many states are not sampled during January and February, and Maine for instance is not sampled in March or April either (full details about non-sampled state-months are available in Supplementary Table S1). We drop these state-months from our estimation samples. The only states for which data is collected in all months for all modes are Florida, Alabama, and North Carolina. There are a total of 35 state-months for which no data is collected for the private boat and shore modes, so data for these modes includes only 785 observations, and 50 state-months have no data collection for the charter mode, leading to 735 observations.

The MRIP data collection process was partially impacted by COVID conditions, however, the main data that we rely on was able to continue unhindered. Specifically, the FES mail survey was able to continue, which is what is necessary for the estimation of effort (trips) in each state-month. The APAIS however was impacted by COVID in that many access points were closed and the survey was unable to be conducted during March and April of 2020. If it were not for this fact, it would be possible to investigate other outcomes such as the mean hours fished on a trip, the average distance traveled to the fishing site, or other attributes of trips which come from the APAIS data.

#### COVID stringency index

Our key independent variable, on state-month level COVID stringency, comes from the Oxford COVID Government Response Tracker project. This data, as described by Hale et al.^17^, is a global dataset tracking government response to COVID across 19 distinct policy indicators such as school- and work-closures, stay-at-home-orders, and movement restrictions. These data are tracked at the daily level, and for the US are available by state. The data have been continuously updated since February 2020 by a team of volunteers who parse government reports, news, and other sources of info to create a standardized set of indicators which are comparable across the globe and across time. The full dataset includes indicators for economic response and health policy, as well as the closure indicators mentioned previously. The open dataset includes links to news articles and other information sources for users to verify the accuracy of the indicator data, and a detailed codebook is available discussing nuances of how globally varied and complex policies were standardized for the purpose of creating the dataset. This dataset has been used in a variety of literature, including evaluating the effect of COVID policies on the spread of the virus^18^, on crime^19^, and indeed also on recreational fishing^12^.

We use the Stringency Index, which combines all 8 closure indicators, as well as a variable indicating the presence of public information campaigns, such as the “flatten the curve” campaign which was common throughout the US circa March and April 2020. All indicators in this index are recorded on discrete, ordinal scales with either 3, 4 or 5 ordinal levels. The Stringency Index is a simple unweighted average of a subindex ranging from 0 to 100 for each indicator. For instance, one of the indicators regards restrictions on internal movement and has 3 ordinal levels, with level 0 being no measures, level 1 being recommendations not to travel between regions or cities, and level 2 being internal movement restrictions. For this indicator, level 0 corresponds to a subindex value of 0, level 1 to 50, and level 2 to 100.

Hallas et al.^20^ present additional information pertinent to the US state-level data, as well as the stylized facts in the data, such as the multiple cycles of stringency at first tightening, followed by loosening policy until a new COVID wave and increased community transmission results in a re-tightening of COVID stringency. Additionally, over time the political gap in Stringency widened with some states having progressively tighter COVID policy compared to other states. For our analysis we calculate the average of the Stingency Index over all days for each state-year-month combination in the dataset.

#### Population data

In order to place the MRIP data on a comparable per-capita basis across states, we include information on state populations using annual population estimates for each state in the sample. For 2017–2019, these are estimates from the American Community Survey, while for 2020 and 2021 we use figures from the Decennial Census, due to inconsistencies in the ACS data for 2020 due to low response rates.

### Descriptive statistics

**Figure 1:**
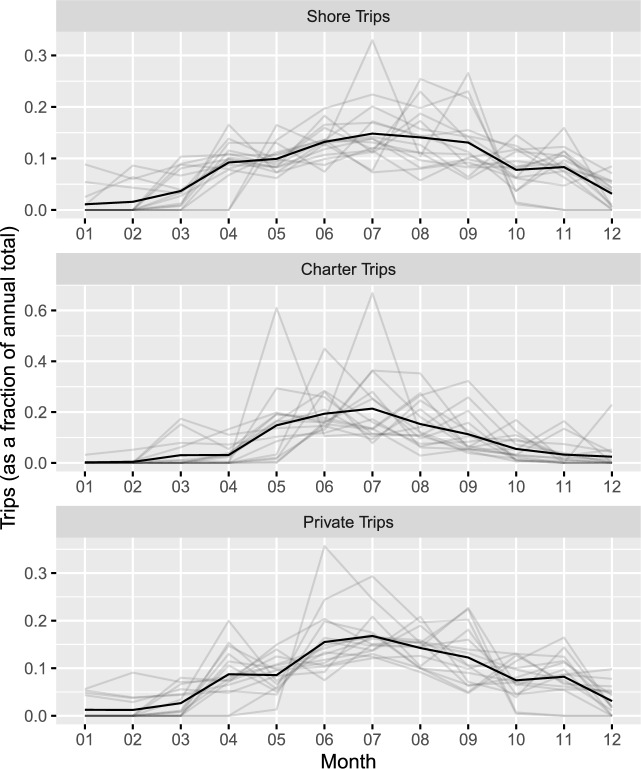
2019 Monthly Marine Sportfishing Trips by State, relative to annual total. Note: The seasonal profile of each state is plotted as a separate grey line, with each month’s value expressed as a percentage of the total annual trips for the state. The bold line gives a simple average of these relative monthly values across the 16 states.

Figure 1 displays seasonal trends in the level of trips across the 16 states in the dataset during 2019, for each mode of fishing. Each grey line plots the seasonal pattern for one state, with the monthly values for each state displayed as a fraction of the annual total. The black line provides a simple average of these relative values across the 16 states within each month, so that the black line represents the composite national-level seasonality trend in fishing trips among these 16 states. July is both the modal peak month among the states, and the peak month for the national trend, for all 3 modes of fishing. There are many state-months in which 0 trips are recorded, or in which no sampling is conducted due to adverse weather conditions, in which case trips can be safely assumed to be near 0, and in particular for the private mode there are only 4 states with positive trips in January: Florida, North Carolina, Alabama, and Mississippi.

We can see from Fig. 1 that fishing activity for the private mode is in most states fairly broadly distributed from April through November, with only 10 state-months accounting for more than 20% of the annual trips in a state, and a maximum of 35% for Maine in June. All but 1 of the 10 state-months with more than 20% of annual trips come from the Northern states of Maine, New Hampshire, Massachusetts, Delaware and Rhode Island, with July in Mississippi as the exception. While the pattern for the shore mode is quite similar to private mode, we can see that the charter mode has substantially more concentration in the Summer, reflecting the fact that those fishing with their own equipment are more likely to get use out of that equipment throughout the year.

**Figure 2:**
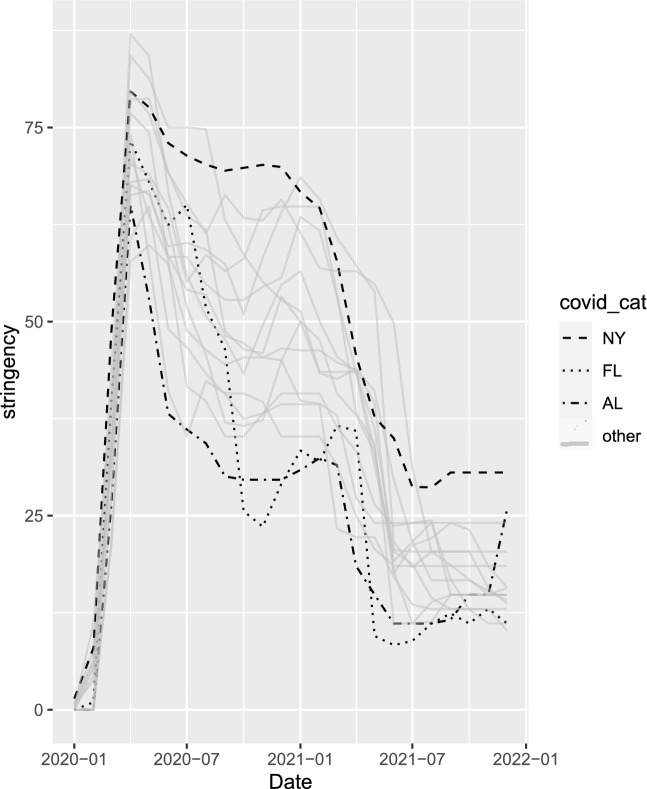
Monthly 2020 stringency Index by State. *Notes*: The legend indicates line patterns for each state, with all other states shown in the solid gray lines. The states highlighted here show a diversity of stringency paths, NY has consistently stringent policy, AL loosens policy substantially after April 2020, while FL displays the most variation in stringency.

Figure 2 shows the evolution of COVID policy stringency over the course of 2020 and 2021 for each of the 16 states in our analysis. Following the initial uniform jumps in stringency during the March/April 2020 lockdowns, there is substantial variation between the states in how COVID policy evolved. Here we have highlighted 3 states showing archetypal cases: (1) New York, which had consistently stringent COVID policy, (2) Alabama, where after April COVID policy was loosened substantially, and stayed loose, and (3) Florida, which displays the most variation in COVID policy over time. The substantial independent variation in these series affords the opportunity to examine the relationship between COVID policy stringency and fishing activity.

### Modeling approach

We model the relationship between the aggregate monthly number of saltwater fishing trips trips taken in each state and the monthly COVID policy stringency in each state using a fixed effect Poisson regression model with the following conditional mean:

$$\begin{aligned} \mu _{imy} = W_{iy}*\exp (\beta _1 S_{imy} + \beta _2 S_{imy}^2 + \alpha _{im} + \gamma _i y) \end{aligned}$$

where for state $i$ during month $m$ and year $y$, $\mu _{imy}$ is the expected aggregate trips, $W_{iy}$ is the state population, $S_{imy}$ is the daily average COVID policy stringency, and we also include the squared term $S^2_{imy}$ to capture a non-linear relationship between stringency and trips taken. The coefficients $\beta _1$ and $\beta _2$ capture the relationship between stringency and trips taken, while the term $\alpha _{im}$ represents state-month fixed effects which allow for state-specific seasonality trends in our model, and $\gamma _i$ is a state-specific linear time trend. The fixed effects Poisson model is efficient and consistent as long as the conditional mean is correctly specified^21^. In other words, even if overdispersion is present in the data, meaning that the conditional variance exceeds the conditional mean, the Poisson model is robust for calculating parameters of the conditional mean. If one desires to calculate conditional probabilities, then overdispersion becomes an issue for inference based on the Poisson model, but we will not be doing so in any of our discussion, we are strictly interested in how stringency influences the mean trips taken. However, for completeness, we also present results based on a Negative Binomial model in Supplementary Table S2.

We calculate variance estimates clustered at the state level, so as to account for unmodelled correlation between the number of trips taken in different periods within a state, as may be the case for instance if unfavorable weather in a given month causes some trips to be postponed to the following month. Furthermore, we adjust the clustered variance estimates to avoid the downward bias that can can occur when the number of clusters is small. Specifically, our variance estimates are calculated using a bias-reduced linearization method^22^. These methods should make inference on our model more robust, though it is worth noting that other phenomena may be present that these techniques do not account for, such as spatial autocorrelation. We estimate the Poisson regression model using the fixest package in R^23^, and variance estimation is conducted through the clubSandwich package in R^24^.

## Results

Table 1 presents the results of our estimated fixed effects Poisson regression models, with the state-specific time trends (of which there are 16) and seasonality fixed effects (of which there are 157) suppressed to focus on the coefficients of interest. In each of the 3 fitted models for the different fishing modes, the first order relationship is an increase in fishing trips at moderate levels of policy stringency, with a second order effect dampening or even reversing this relationship at higher levels of stringency. The first order relationships are significant at the 1% level for private mode trips, and at the 5% level for shore mode, while the coefficients for the charter mode are significant only at the 10% level. The second order coefficient is significant only for the private mode, though it has a negative sign in all three models.

**Table 1:** Poisson fixed effect regression of trips on Covid-19 stringency, by mode.

	Private	Charter	Shore
Stringency	$$0.0135^{***}$$	0.0236	$$0.0172^{*}$$
	(6.61)	(1.88)	(2.43)
Stringency$$^2$$	$$-0.0002^{***}$$	$$-0.0004$$	$$-0.0002$$
	$(-4.94)$	$(-2.09)$	$(-2.08)$
Num. obs.	785	735	785
Pseudo R$^2$	0.9578	0.8939	0.9356
Log Likelihood	$-9,257,548.6$	$-438,983.7$	$-19,966,491.8$
BIC	18, 516, 363.6	879, 155.5	39, 934, 250.1

$$^{***} \textit{p}<0.001$$; $$^{**} \textit{p}<0.01$$; $$^{*} \textit{p}<0.05$$. t-statistics in parentheses calculated using standard errors clustered by state with small-sample adjustment

**Figure 3:**
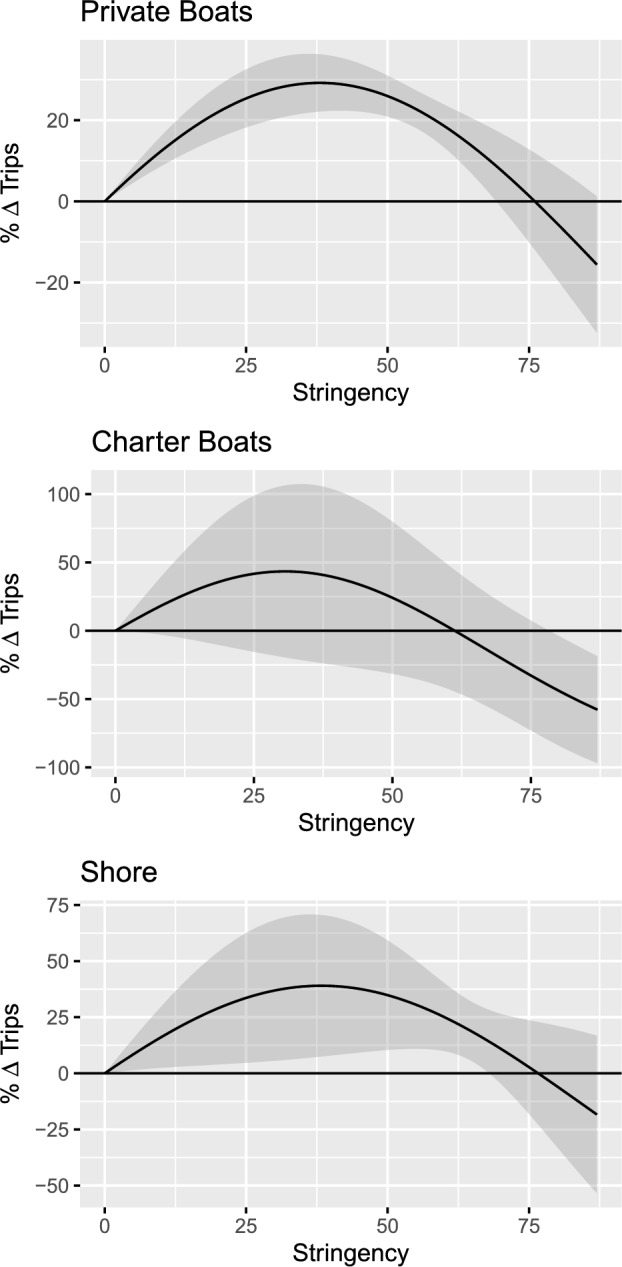
Predicted percentage change in trips as a function of the stringency index.

Figure 3 represents these same relationships graphically, by plotting the estimated model’s predictions for the relative change in resident fishing trips at the various possible levels of COVID stringency. The solid line provides the point estimates for predicted percentage change in trips, while the shaded areas plot the 95% confidence intervals around these predictions.

For the private mode, we can see that the “break even” point in Fig. 3 occurs at a stringency score of approximately 75. Referring back to Fig. 2, we can see that this level of stringency was reached only in March and April of 2020 for some states. Therefore, for most of the observations in the dataset, stringency is at intermediate levels for which our model predicts increases in the level of private fishing activity. Similar break-even points apply for the charter and shore modes, although these are estimated with less precision as illustrated by the wider confidence intervals in Fig. 3.

## Conclusion

In this paper, we measure the relationship between COVID policy stringency and recreational marine fishing effort, among 16 states in the U.S. east coast and Gulf of Mexico. To do so, we rely on data from the MRIP to measure the total number of angler-trips in each state-month, and on the OxCGRT dataset to measure the daily average level of COVID policy stringency in each state-month. We use a fixed effect Poisson model to estimate the relationship between COVID policy stringency and total angler-trips taken, allowing for state-specific seasonality trends and time trends in the number of fishing trips.

We find that the aggregate number of marine fishing trips increases at a decreasing rate with COVID policy stringency and suggest that this inverse-U relationship is the outcome of countervailing forces: (1) stringency reduces available substitutes of indoor or high-density outdoor activities, increasing the attractiveness of fishing, which is the dominant force on the low-stringency side of the curve, and (2) COVID risk makes even a relatively distanced activity, such as fishing, less appealing compared to entirely in-home activities such as cooking, backyard games, video games, etc., which in addition to site closures is the dominating force on the high-stringency side of the curve.

Overall, there was an approximately 20% increase in private boat trips taken at the levels of COVID stringency which predominated in most states by late 2020 into 2021, suggesting that marine fishing activity was fairly stable during this period, relative to socioeconomic indicators like unemployment, GDP, etc. which saw large fluctuations^25^.

It is important to note that these results for the impact of COVID stringency on fishing trips represent a composite effect of factors correlated with COVID stringency. For instance, to the degree that traffic lessened in proportion to stringency, the travel cost to visit fishing sites was reduced and therefore our results are partially driven by that change. Similar logic would apply for other correlated factors, including for instance unemployment rates, which both increase the leisure time available for fishing and reduce the disposable income available for fishing.

In interpreting these results, one consideration is that the estimated increase in trips at moderate levels of policy stringency may reflect the dynamic behavior of anglers. That is, since the periods of moderate stringency were immediately preceded by periods of high stringency, the increase in trips may be due to anglers saving up time and money for going on trips when stringency was high (for further details on excess household savings during the pandemic see Aladangady et al.^26^), and additionally having an increased desire to go fishing after having been unable or unwilling to go fishing during the high stringency period. Our data do not provide sufficient resolution to explicitly model such intertemporal substitution dynamics, which would be best modeled at the level of individual anglers if individual-level panel data on fishing activity were available, but nonetheless these dynamics are a plausible explanation for the trend observed in the aggregate data.

This paper has considered specifically the impact of state-level COVID stringency on in-state marine fishing trips taken by residents. Future research may also consider the question of cross-state substitution of fishing trips, a question which can not be answered sufficiently with the MRIP data we rely on in this paper. Such cross-state substitution may be due to anglers seeking places with lower COVID stringency to vacation, or perhaps also by an increase in mobility due to the rise of remote work arrangements (see Whitaker^27^ for more on the issue of internal migration during COVID). One data source which may be appropriate to addressing this question is the National Saltwater Angler Registry (NSAR), a database of all marine fishing license sales for states covered by the MRIP program. The NSAR database contains sufficient information to uniquely identify anglers, and could therefore be used to measure not only the rate of sales of out-of-state temporary licenses to anglers from various origin states, but also the rate of migration of anglers from one state to another based on where they have purchased in-state licenses. Such analysis would likely show an increase in angler migration to major destinations like Florida during COVID, but what is less clear is whether a general pattern of migration toward states with lower COVID stringency would be observed, such as from New York to New Hampshire.

In future research, it may be possible to assess the impact of COVID on fishing activity in different ways. For instance, one data source that could be utilized is foot traffic data based on cellphone GPS tracking, as has already been used to assess outdoor recreation activity during the pandemic in other contexts^28^. In this way it would be possible to measure the level of activity near the coastline on a much finer spatial and temporal scale than is possible with the survey data we rely on in this paper. It would be a challenge to distinguish between activities such as beach going and other uses of the coastline versus fishing-specific activity with this data source, and additionally it would likely not be possible to assess boat-based activity, however the advantages in terms of data resolution may still make it an attractive option.

In regards to policy implications, our results may be interpreted in a few ways. For instance, to the extent that our results reflect the increased internal migration within the US, perhaps certain states in the Southeast will need to take account of persistent increased fishing pressure, while other states that have lost population will not. Or, to the extent that the results are driven by new participants in fishing, it will have to be seen whether these new participants continue to use the fishery before we know if there will be long-run ramifications for management. Relatedly, if increases in congestion (eg. boat ramps being busier, not only due to increased fishing activity but also increased boating in general) have effectively increased the cost of fishing trips, then angler welfare per trip may be reduced, which would have implications for the management of the fishery. Future research may seek to better understand the determinants of the change in effort, as well as the implications of these changes for angler welfare and net benefits from the fishery.

## Supplementary Information

Supplementary Information.

## Data Availability

All data used in this analysis are public use datasets, available directly from their respective sources. For convenience, copies of the data as used in our analysis, along with accompanying analysis code to replicate the results in paper, are available on Github (primary final merged dataset is tripsTMPS.rds, which is a binary data file that can be opened with R software using the readRDS() function): https://github.com/zander-gordan-noaa/covidRecreation
