# Supplementary material for: Resident marine sportfishing effort in the United States varied non-monotonically with COVID policy stringency
Source: Sci Rep. 2024 May 29;14:12332. doi: 10.1038/s41598-024-60960-4 (PMC11634898; doi:10.1038/s41598-024-60960-4)
Supplement: Supplementary file 1 — Supplementary Information. [file 41598_2024_60960_MOESM1_ESM.pdf]

Supplementary Information for "Resident Marine Sportfishing Effort in the United States Varied Non-Monotonically with COVID Policy Stringency"

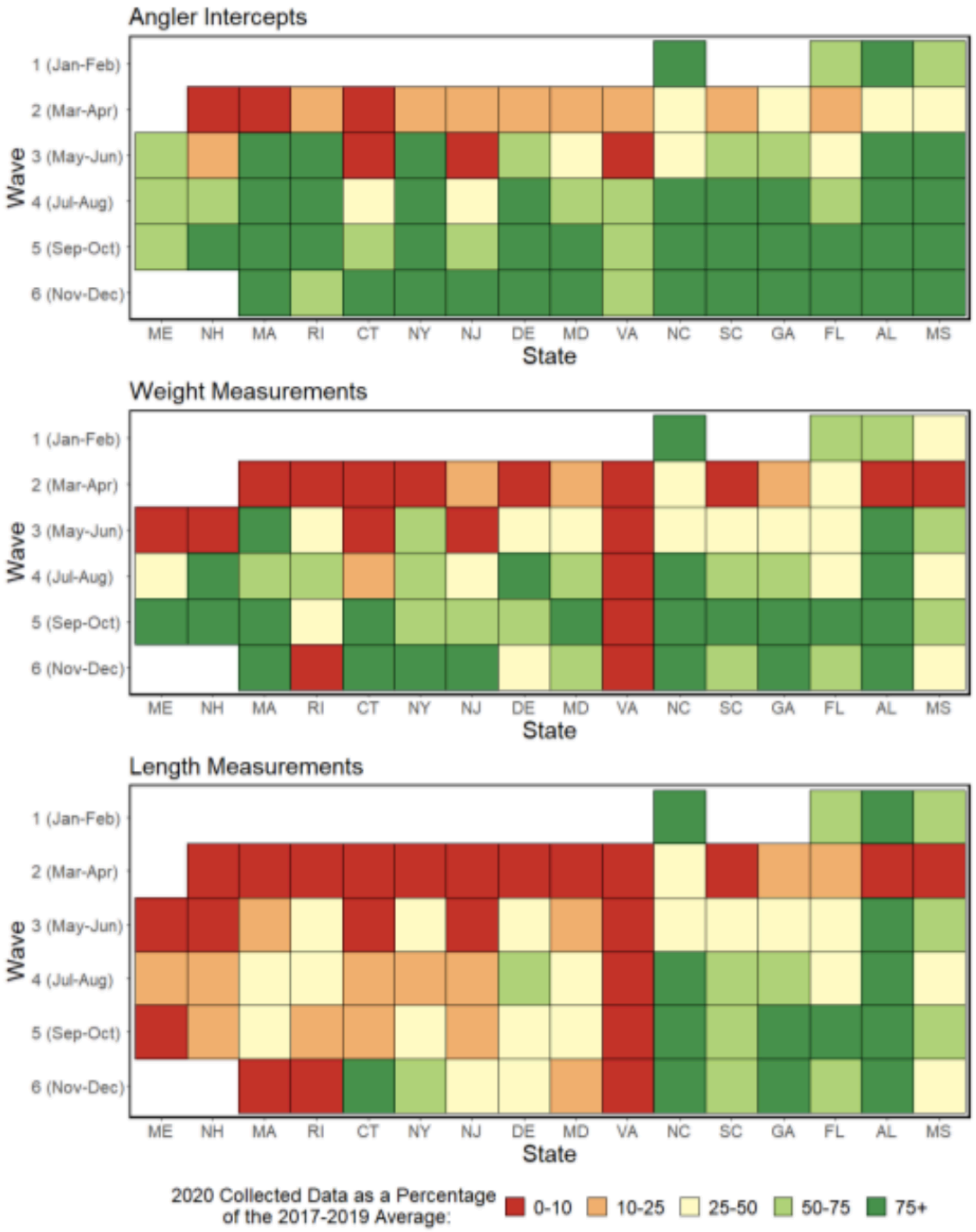

Supplementary Figure S1: The white squares indicate state-months in which the MRIP program does not collect data due to low levels of fishing activity. Source: MRIP Survey Design Manual.

	Private	Charter	Shore
Stringency	0.0158	0.0476	0.0076
	(2.27)	(3.39)	(1.23)
Stringency^2	-0.0002	-0.0006	-0.0001
	(-2.61)	(-3.24)	(-1.50)
Num. obs.	785	735	785
Log Likelihood	$-10,033.9$	$-6,027.1$	$-10,364.1$

Supplementary Table S2: Negative Binomial Fixed Effect Regression of Trips on Covid-19 Stringency, by Mode. Compare with Table 1 in the main paper. Note that the regression specification is slightly different in this table because it is not possible to have varying slopes in the Negative Binomial version of the fixed-effects R package we use, so rather than having a state-specific time trend, there is just one overall time trend. Additionally, variance estimation is done in the standard fashion because the state-clustered standard errors with small-sample adjustment that we use in the Poisson model are not compatible with the Negative Binomial.

References

Bergé L (2018). “Efficient estimation of maximum likelihood models with multiple fixed-effects: the R package FENmlm.” *CREA Discussion Papers*.

National Marine Fisheries Service Office of Science and Technology. 2023. Marine Recreational Information Program Survey Design and Statistical Methods for Estimation of Recreational Fisheries Catch and Effort. Silver Spring, MD.
